# Phosphate Removal in Relation to Structural Development of Humic Acid-Iron Coprecipitates

**DOI:** 10.1038/s41598-018-28568-7

**Published:** 2018-07-09

**Authors:** Kai-Yue Chen, Liang-Ching Hsu, Ya-Ting Chan, Yen-Lin Cho, Fang-Yu Tsao, Yu-Min Tzou, Yi-Cheng Hsieh, Yu-Ting Liu

**Affiliations:** 1 Department of Soil and Environmental Sciences, National Chung Hsing University, 145 Xingda Rd., Taichung, 40227 Taiwan; 2 Scientific Research Division, National Synchrotron Radiation Research Center, 101 Hsin-Ann Rd., Hsinchu, 30043 Taiwan; 3 Innovation and Development Center of Sustainable Agriculture, National Chung-Hsing University, 145 Xingda Rd., Taichung, 40227 Taiwan; 4 Office of the Texas State Chemist, Texas A&M AgriLife Research, Texas A&M University System, College Station, TX 77843 USA

## Abstract

Precipitation of Fe-hydroxide (FH) critically influences the sequestration of PO_4_ and organic matter (OM). While coatings of pre-sorbed OM block FH surfaces and decrease the PO_4_ adsorption capacity, little is known about how OM/Fe coprecipitation influences the PO_4_ adsorption. We aimed to determine the PO_4_ adsorption behaviors on humic acid (HA)-Fe coprecipitates in relation to surface and structural characteristics as affected by HA types and C/(C + Fe) ratios using the Fe and P X-ray absorption spectroscopy. With increasing C/(C + Fe) ratios, the indiscernible changes in the proportion of near-surface C for coprecipitates containing HA enriched in polar functional groups implied a relatively homogeneous distribution between C and Fe domains. Wherein PO_4_ adsorbed on FH dominated the P inventory on coprecipitates, yielding PO_4_ sorption properties nearly equivalent to that of pure FH. Structural disruptions of FH caused by highly associations with polar functional groups of HA enhanced the C solubilisation. While polar functional groups were limited, coprecipitates consisted of core FH with surface outgrowth of HA. Although surface-attached HA that was vulnerable to solubilisation provided alternatively sites for PO_4_ via ternary complex formation with Fe bridges, it also blocked FH surfaces, leading to a decrease in PO_4_ adsorption.

## Introduction

Excess phosphate (PO_4_) originated from over fertilizer applications and industrial activities such as optoelectronic industry has caused nonpoint and point source pollutions, leading to the eutrophication and hypoxia in aquatic systems^1^. Compared with 4–12 mg L^−1^ of total phosphorus (TP) in municipal wastewater^2^, up to 500 mg L^−1^ of TP is discharged from the optoelectronic industry^3^, exceeding effluent TP regulations in the USA (0.07–0.1 mg L^−1^)^4^ and Europe (1–2 mg L^−1^)^5^. Phosphate in wastewater could be removed in processes of coagulant addition via chemical precipitation, coagulation of particulate forms, and adsorption onto chemical flocs of iron (Fe)/aluminum (Al) hydroxides^6^. The enhanced coagulation with hydrolyzed Al or Fe has also be performed to eliminate dissolved organic matter (DOM) in drinking water treatment^7^ as DOM could serve as precursors for carcinogenic chlorination by-products^8^. When Al and Fe coagulants are added to water, hydrolysis products are able to destabilize the DOM via charge neutralization, enmeshment, and adsorption^9^. The optimum coagulant concentration (OCC), i.e. the lowest dosage to reach the maximum decrease in turbidity, for ferric chloride (FeCl_3_) was suggested from 2.4  ${\rm{\times }}$ 10^−4^ to 7.7  ${\rm{\times }}$ 10^−4^ mol L^−1^ at pH 6-8 in systems containing 15–20 mg L^−1^ of dissolved organic carbon (DOC)^10,11^. However, only about 20% of DOC was removed just before the OCC; then DOC diminished with increasing FeCl_3_ addition to reach the maximum of 40% removal at [Fe] = 2.0  ${\rm{\times }}$ 10^−3^ − 2.5  ${\rm{\times }}$ 10^−3^ mol L^−1^ (molar C/Fe ratio = 0.5–0.6). As FeCl_3_ dosage was increased up to 3.0  ${\rm{\times }}$ 10^−3^ mol L^−1^ (molar C/Fe ratio = 0.4), DOC was again released back to the suspensions^10^.

Recent Fe K-edge extended X-Ray absorption fine structure (EXAFS) studies have reported that hydrolyzed Fe was poorly polymerized in the presence of DOM around OCC even at basic pH^10,12^. At the dosage higher than OCC, the sweep flocculation occurred and Fe species was similar to that precipitated during the neutralization of Fe salts such as ferrihydrite^10^. Such non- and poorly-crystalline Fe-hydroxides generated are widely distributed in aquatic environments^13^. On account of ubiquitous DOM in surface waters, Fe-hydroxides and DOM are likely associated into complex assemblages^14–17^. While coprecipitated with DOM, Fe-hydroxides showed the variety in particle size, specific surface areas, structural crystallinity, and consequently the reactivity for PO_4_ adsorption^14,15,18,19^. Understanding the interactions between DOM and Fe-hydroxides is critical as subsequent changes in surface chemistry significantly influence the complexation state of DOM-Fe nanoparticles as well as the fate and dynamics of the concomitant dissolved species such as PO_4_^20,21^.

Humic acid (HA) with colloidal natures whose size and charge distributions are controlled by functional groups^19^ was used as DOM models here. Coprecipitations with two types of HA were tested as differences in DOM composition might affect the DOM/Fe association^13,16^, and thereby the PO_4_ adsorption behaviors. In this study, the coprecipitation between two types of HA and Fe was performed in molar C/(C + Fe) ratios from 0.17 to 0.33, corresponding to molar C/Fe ratios of 0.4 to 0.5 wherein the DOC was restabilized from the greatest degree of destabilization in the presence of Fe coagulants^10^. We aimed to (1) examine at a molecular scale surface and structural characteristics of coprecipitates as affected by HA types and C/(C + Fe) molar ratios using Fe K-edge EXAFS, X-ray photoelectron spectroscopy (XPS) and other techniques, and (2) determine how such attributes affect PO_4_ adsorption capacity in relation to P speciation derived from the P K-edge X-ray absorption near edge structure (XANES) spectroscopy.

## Results

### Characteristics of YHA and AHA

Results of the elemental analysis showed a comparable composition of C, H, and O in the Yangming humic acid, (YHA) and Aldrich humic acid (AHA), although the relative proportion of N in the YHA was nearly three times higher than that in AHA (see Supplementary Table S1). Integrative results of ^13^C NMR data suggested that the total proportion of polar groups including *O*-alky C, phenolic C, carboxyl C, and carbonyl C for the YHA and AHA samples were 58.0 and 49.8% (see Supplementary Table S1). Wherein, the proportion of *O*-alky C and carboxyl C in YHA was 2.2 and 1.4 times greater than that in AHA. In addition, the relatively intensified signal in the 170–180 ppm region of YHA also implied a greater proportion of amide groups in YHA than that in AHA (see Supplementary Fig. S1)^22^.

**Figure 1:**
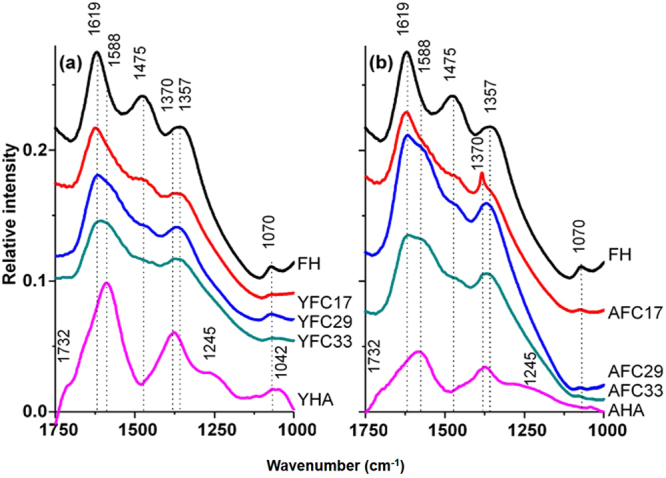
FTIR spectra of Fe-hydroxide (FH), humic acid (YHA and AHA), and HA/Fe coprecipitates containing (**a**) YHA and (**b**) AHA with initial C/(C + Fe) ratio of 0.17, 0.29, ad 0.33 (YFC17/29/33 and AFC17/29/33).

In terms of FTIR results, both YHA and AHA contained functional groups of –C=O stretching of –COOH, asymmetric –COOH stretching of carboxyl groups, symmetric –COO^−^ stretching of –COOH, and –OH stretching of phenolic OH as indicated by peaks at 1732, 1588, 1370, and 1245 cm^−1^ (Fig. 1)^23,24^. The peak at 1245 cm^−1^ might also be contributed by the OH deformation of –COOH^23,25^. In addition, N-H bending of amine group also showed signals between 1650 and 1580 cm^−1 ^^26,27^. For the peak at 1042 cm^−1^ found in YHA but not in AHA, it could be assigned to –CO stretch of carbohydrates^23,24,28^ and/or the amine –CN stretch^26,27^. Collectively, the ^13^C NMR and FTIR results proposed that YHA was relatively enriched by polar functional groups, practically the carboxyl and amide/amine groups. The greater proportion of amide/amine groups was paralleled by the 2.9-fold higher N composition in YHA than in AHA (see Supplementary Table S1).

**Figure 2:**
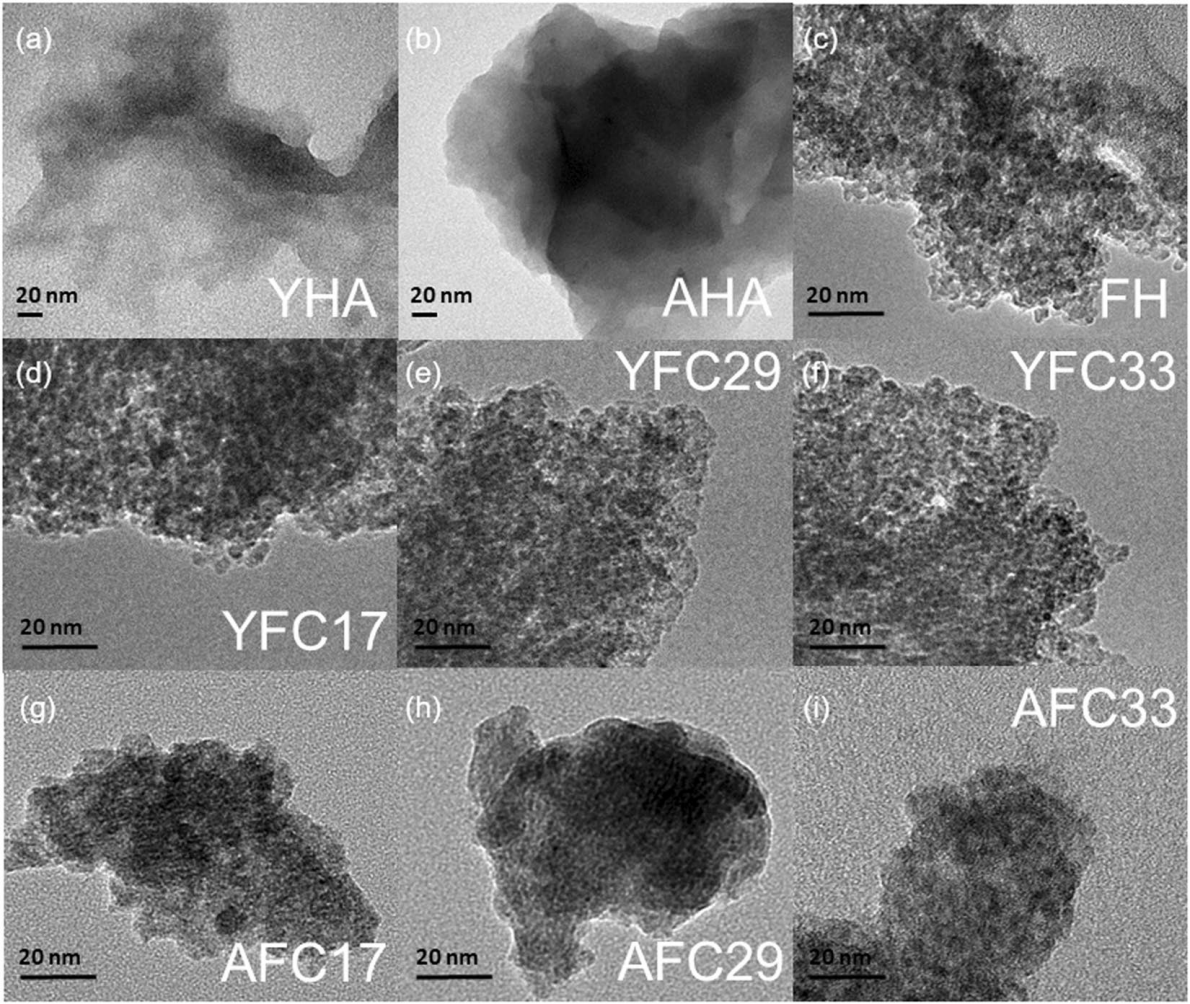
TEM images for humic acid (YHA and AHA), Fe-hydroxide (FH), and HA/Fe coprecipitates containing YHA and AHA with initial C/(C + Fe) ratio of 0.17, 0.29, and 0.33 (YFC17/29/33 and AFC17/29/33).

Regarding morphologies, TEM micrographs in Fig. 2a,b showed the sponge-like structures for YHA and AHA. Compared with the well-delimited clusters for AHA, humic networks of YHA presented as polydisperse domains with less degree of aggregation, agreeing with the greater proportion of polar functional groups, whose negatively charges caused the electrostatic repulsion and inhibited the aggregation of YHA^13^.

### Precipitation, re-solubilisation, and distribution of C and Fe for HA/Fe coprecipitates

The HA/Fe coprecipitates containing YHA and AHA were denoted as YFC17/29/33 and AFC17/29/33 hereafter. Compared to AFC samples, greater amounts of C precipitated in YFC samples led to the 1.13~1.30-fold higher precipitated C/(C + Fe) molar ratios (Table 1). Previous studies found that the degree of C coprecipitation with Fe(III) depended on the composition of organic matter (OM)^15,16^. While the OM formed insoluble complexes with Fe(III) during coprecipitation, the solid products might contain greater contents of C than the nanoparticles of Fe-hydroxides with adsorbed OM^15^. Thus, our results could probably be attributed to the enrichment of polar functional groups in YHA that facilitated the complexation between Fe and C and subsequently the C precipitation for YFC samples^15,29^.

**Table 1:** Ratios, concentrations, and solubilisation of C and Fe for HA/Fe coprecipitates containing humic acid (YHA and AHA) with initial C/(C + Fe) ratio of 0.17, 0.29, and 0.33 (YFC17/29/33 and AFC17/29/33)^a^.

Sample	Initial C/(C + Fe)	Precipitated C (mol kg^-1^)	Precipitated Fe (mol kg^-1^)	Precipitated (bulk) C/(C + Fe)	C solubilisation (%)	Fe solubilisation (%)
YFC17	0.17	2.53 (0.04)	10.31 (0.08)	0.20 (0.003)	9.68 (2.18)	N.D.^b^
YFC29	0.29	4.00 (0.09)	9.98 (0.02)	0.29 (0.007)	5.41 (0.20)	N.D.
YFC33	0.33	5.75 (0.07)	9.48 (0.05)	0.38 (0.005)	3.99 (0.74)	N.D.
AFC17	0.17	2.17 (0.04)	10.30 (0.07)	0.17 (0.003)	19.9 (0.70)	N.D.
AFC29	0.29	2.67 (0.05)	9.44 (0.07)	0.22 (0.004)	7.04 (0.00)	N.D.
AFC33	0.33	4.83 (0.02)	9.69 (0.00)	0.33 (0.002)	5.00 (0.11)	N.D.

^a^Numbers in parentheses represent standard deviations derived from three replicates.

^b^Not detected.

The stabilization of HA/Fe coprecipitates was determined based on the C and Fe solubilisation upon a 42-h incubation in the 0.01 M KCl solution. While no discernible Fe release was found for all samples, up to 9.68% of C was released from the YFC17, and the proportion decreased to 3.99% for YFC33. Carbon solubilisation of AFC samples also decreased with increasing C/(C + Fe) ratios but with greater proportion than the YFC samples even at the same initial C/(C + Fe) ratio (Table 1). These results suggested that the structural stabilization of HA/Fe coprecipitates might be subject to HA types and precipitated C/(C + Fe) ratios.

**Figure 3:**
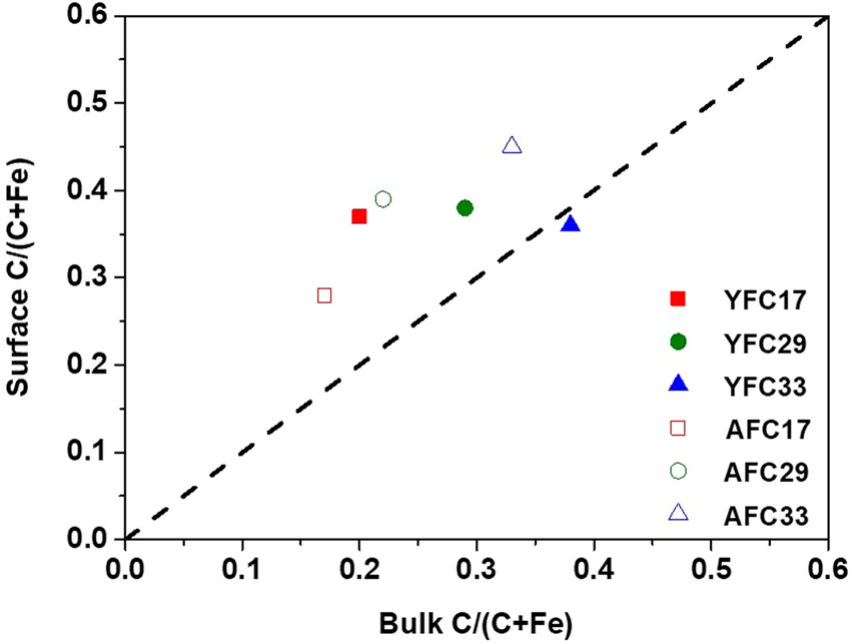
The relationship between C/(C + Fe) ratio near coprecipitate surfaces and that in bulk samples for HA/Fe coprecipitates containing humic acid (YHA and AHA) with initial C/(C + Fe) ratio of 0.17, 0.29, and 0.33 (YFC17/29/33 and AFC17/29/33).

According to XPS results, surface C/(C + Fe) ratios for all samples were generally greater than that in bulk samples, suggesting that C dominated coprecipitate surfaces (Fig. 3). However, different trends in C distribution on YFC and AFC samples implied the distinct interactions between HA and Fe domains. While the surface C/(C + Fe) ratios of AFC samples increased from 0.28 to 0.45 with increasing bulk C/(C + Fe) ratios from 0.17 to 0.33, that of YFC samples remained nearly constant (0.36–0.38, Fig. 3), irrespective of the bulk C/(C + Fe) ratios. These results indicated a 1.4~1.7 fold enrichment of near-surface C relative to total C in AFC samples (Fig. 3). Such narrow range in the ratio between surface and bulk C implied an analogous interaction mechanism between AHA and Fe nanoparticles, regardless of the added C/(C + Fe) ratios. In contrast, the ratios of surface C to bulk C in YFC17, YFC29, and YFC33 coprecipitates were 1.9, 1.3, and 1.0, respectively. The increasing consistency between near-surface and bulk C with increasing C proportion for YFC samples could be plausibly caused by the increasing particle segregation, increasing OM domain size and/or decreasing Fe-hydroxide domain size. Such significant discrepancies in the C distribution implied substantial changes in the structural association between C and Fe caused by different HAs.

### Functional groups of Fe-hydroxides and HA/Fe coprecipitates

The FTIR spectrum of FH in Fig. 1 showed the contribution of adsorbed water at 1619 cm^−1^^30^, asymmetric stretch of carbonate at 1475 cm^−1 ^^31^, the Fe-O band and/or symmetric stretch of carbonate at 1357 cm^−1^ and 1070 cm^−1 ^^31–33^. All YFC samples showed similar FTIR spectra to that of FH, but with less pronounced peaks (Fig. 1a). With increasing C/(C + Fe) molar ratios, the 1619-cm^−1^ band of YFC samples became broad and accompanied the appearance of the shoulder near 1588 cm^−1^. Chen *et al*.^15^ reported that the combination of the emergence for the band near 1615 cm^−1^ in OM/Fe coprecipitates and the decrease in the asymmetric carboxyl band near 1595 cm^−1^ with decreasing C/Fe ratios indicated the strong bonding between asymmetric carboxyl C functional groups and Fe(III). Compared with the symmetric COO^−^ band at 1370 cm^−1^ in YHA, the spectral centroid tended to shift toward the lower wavenumber of 1357 cm^−1^ for the YFC samples (Fig. 1a). Previous studies suggested that this shift might be caused by the ligand exchange between carboxylate and metals^25,34^. However, sample dilution with FH that showed the symmetric COO^-^ band at 1357 cm^−1^ might mask the 1370 cm^−1^ signal from YHA, if present.

In contrast with YFC samples, whose FTIR spectra mimicked that of FH, FTIR spectra of AFC samples were more like that of AHA (Fig. 1b). For example, the symmetric –COO^−^ band of the AFC17 sample occurred at the same wavenumber (1370 cm^−1^) with AHA. This 1370-cm^−1^ band was also found in AFC29 and AFC33, resulting in a broad peak centered at 1367 cm^−1^. Moreover, the emergence of the shoulder at 1588 cm^−1^ in AFC samples with the initial C/(C + Fe) molar ratio ≥ 0.29 evidenced the increasing association between AHA and Fe-hydroxides and the importance of AHA upon the distribution of functional groups on AFC samples.

### Morphologies and structural characteristics of HA/Fe coprecipitates

Transmission electron micrographs showed that the degree of aggregation for the YFC17, YFC29, and YFC33 samples was similar to that of FH, with nanoparticles discernible within the more compact aggregates (Fig. 2c–i). However, morphologies of AFC samples showed a loosely associated aggregation with indistinct nanoaggregates.

The XRD patterns of FH, YFC, and AFC samples showed two broad peaks centered at 2.6 and 1.5 Å (see Supplementary Fig. S2), consistent with the (110) and (300) planes of two-line ferrihydrite^35^. In spite of the subtle changes, the full width at half maximum (FWHM) for the (110) and (300) peaks of the AFC samples tended to broaden systematically with increasing C/(C + Fe) ratios (see Supplementary Fig. S3a and b), implying an increase of stacking disorder of structural layers. For YFC samples, however, the broadest (110) and (300) XRD peaks were found in the YFC17 sample (see Supplementary Fig. S3a and b). Such results might plausibly suggested the smallest scattering domains or the most disrupted ordering of Fe-hydroxide structures in coprecipitates containing the least amount of YHA in this study^36^.

**Figure 4:**
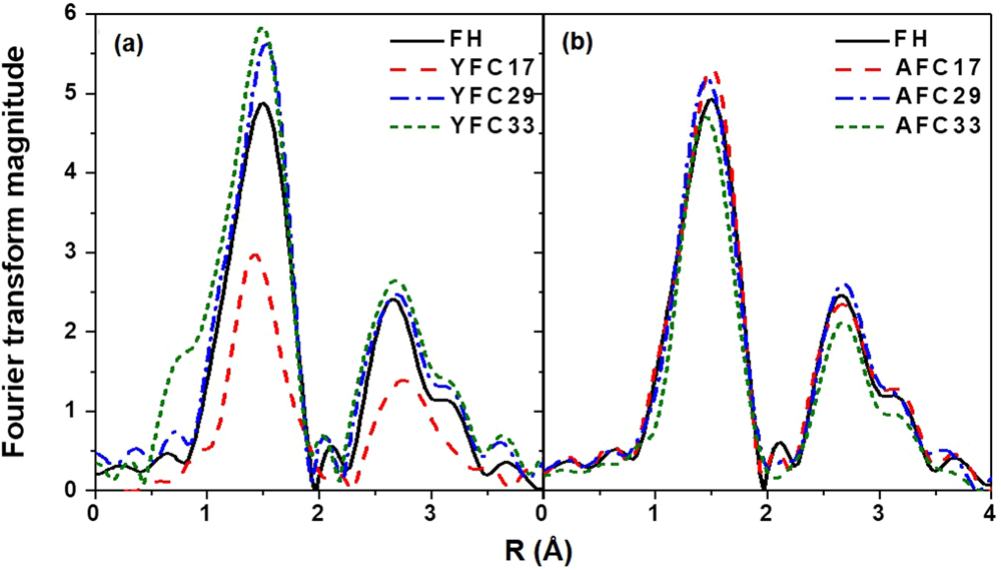
Fourier transformed Fe EXAFS data for Fe-hydroxide (FH) and HA/Fe coprecipitates containing humic acid: (**a**) YHA and (**b**) AHA with initial C/(C + Fe) ratio of 0.17, 0.29, and 0.33 (YFC17/29/33 and AFC17/29/33).

Regarding the Fe EXAFS data, backscattering signals of YFC17 were significantly weaker than that of FH and other HA/Fe coprecipitates (Fig. 4). Modeling of the first-shell Fe-O coordination with six surrounding O atoms across all coprecipitates showed the interatomic distances at 1.97–1.99 Å with the fitted σ^2^ (mean-square displacements of interatomic distances) value of 0.022 and 0.013 for YFC17 and other coprecipitates (Table 2). The relatively greater σ^2^ value possibly explained the shorter FT magnitude peak between 0.8 and 2.0 Å in YFC17 (Fig. 4a). Interatomic distances of Fe-Fe1 and Fe-Fe2 paths were fitted at 3.05–3.07 and 3.43–3.45 Å, corresponding to edge- and corner-shared FeO_6_ octahedra, respectively^37,38^. Of individual AFC samples, the fitted coordination numbers (CN) for Fe-Fe1 and Fe-Fe2 paths showed insignificant differences (outside of model parameter uncertainties) from FH that consists of 4.3 (${\rm{\pm }}$0.7) Fe atoms at 3.07 Å (Fe-Fe1) and 3.2 (${\rm{\pm }}$0.9) Fe atoms at 3.45 Å (Fe-Fe2). The similar trend was also found in the YFC29 and YFC33 samples. However, for the YFC17 sample, the CN for Fe-Fe1 and Fe-Fe2 paths were 1.5 (${\rm{\pm }}$0.7) and 1.3 (${\rm{\pm }}$0.9) (Table 2), which were substantially less than that of FH. Such decrease in CN of Fe at Fe-Fe1 and Fe-Fe2 paths accounted for the notable decrease in the FT magnitude between 2.2 and 3.5 Å (Fig. 4a). The analogous coordination environments of Fe-hydroxide domains among FH and HA/Fe coprecipitates except YFC17 resulted in the nearly identical EXAFS spectra (Fig. 4).

**Table 2:** Fits to Fe EXAFS data for Fe-hydroxide (FH) and HA/Fe coprecipitates containing humic acid (YHA and AHA) with initial C/(C + Fe) ratio of 0.17, 0.29, and 0.33 (YFC17/29/33 and AFC17/29/33)^a^.

sample	Fe-Fe1^b^		Fe-Fe2^b^	
	R (Å)	CN	R (Å)	CN
FH	3.07 (0.01)	4.3 (0.7)	3.45 (0.01)	3.2 (0.9)
YFC17	3.05 (0.02)	1.5 (0.7)	3.43 (0.03)	1.3 (0.9)
YFC29	3.07 (0.01)	4.5 (0.8)	3.45 (0.01)	3.5 (1.0)
YFC33	3.07 (0.02)	4.7 (1.7)	3.45 (0.02)	3.6 (2.0)
AFC17	3.07 (0.01)	4.2 (0.6)	3.45 (0.01)	3.6 (0.7)
AFC29	3.07 (0.01)	4.5 (0.7)	3.45 (0.01)	3.3 (1.0)
AFC33	3.07 (0.02)	3.9 (0.8)	3.45 (0.02)	2.8 (1.0)

^a^Fitting was done across the *k* range of 2.5 to 11.5 Å^−1^ and an R range of 1.0 to 3.5 Å. Numbers in parentheses are uncertainties calculated for the EXAFS model. Paths used in EXAFS fitting are all single-scattering paths. All samples were fit simultaneously, yielding a normalized sum of squared residuals [*R-factor* = Σ(data-fit)^2^/Σdata^2^) of 0.0064 (0.64%)]. S0^2^ (fixed amplitude reduction factor based on first-shell fitting of hematite) = 0.83. ΔE (fitted energy shift parameter) = −1.49 (0.35) eV for all samples. The fitted R (interatomic distance) of Fe-O path across all samples was 1.97–1.99 Å. The CN (coordination number) for the Fe-O path was fixed at 6.0. With the exception of YFC17, the fitted σ^2^ (mean-square displacements of interatomic distances) of the Fe-O path for all samples was 0.013 (0.003) Å^2^. The fitted σ^2^ value of the Fe-O path in YFC17 was 0.022 (0.002) Å^2^.

^b^The fitted σ^2^ values of the Fe-Fe1 and Fe-Fe2 path in YFC17 were both 0.013 (0.002) Å^2^, which for theFe-Fe1 and Fe-Fe2 path in other samples were 0.017 (0.002) and 0.016 (0.002) Å^2^, respectively.

Collectively, Fe-hydroxide domains of the YFC17 sample showed distinct attributes from other YFC and AFC samples. For the YFC17 sample, the relatively greater σ^2^ value of the Fe-O path was suggestive of a more disordered structural development, and the relatively less CN of Fe at Fe-Fe1 and Fe-Fe2 paths implied the disruption in edge- and corner-shard FeO_6_ octahedral linkages^39^. For such nanometer-sized particles (Fig. 2), the decrease in octahedral linkages could be translated to a decrease in the domain size as the result of increasing proportion of terminal octahedra on particles surfaces^36,40^. This result was in light with the broadest diffraction peaks of XRD data in the YFC17 sample, which indicated a most disordered short-range structure (see Supplementary Fig. S2).

### Phosphate sorption isotherms on HA/Fe coprecipitates

**Figure 5:**
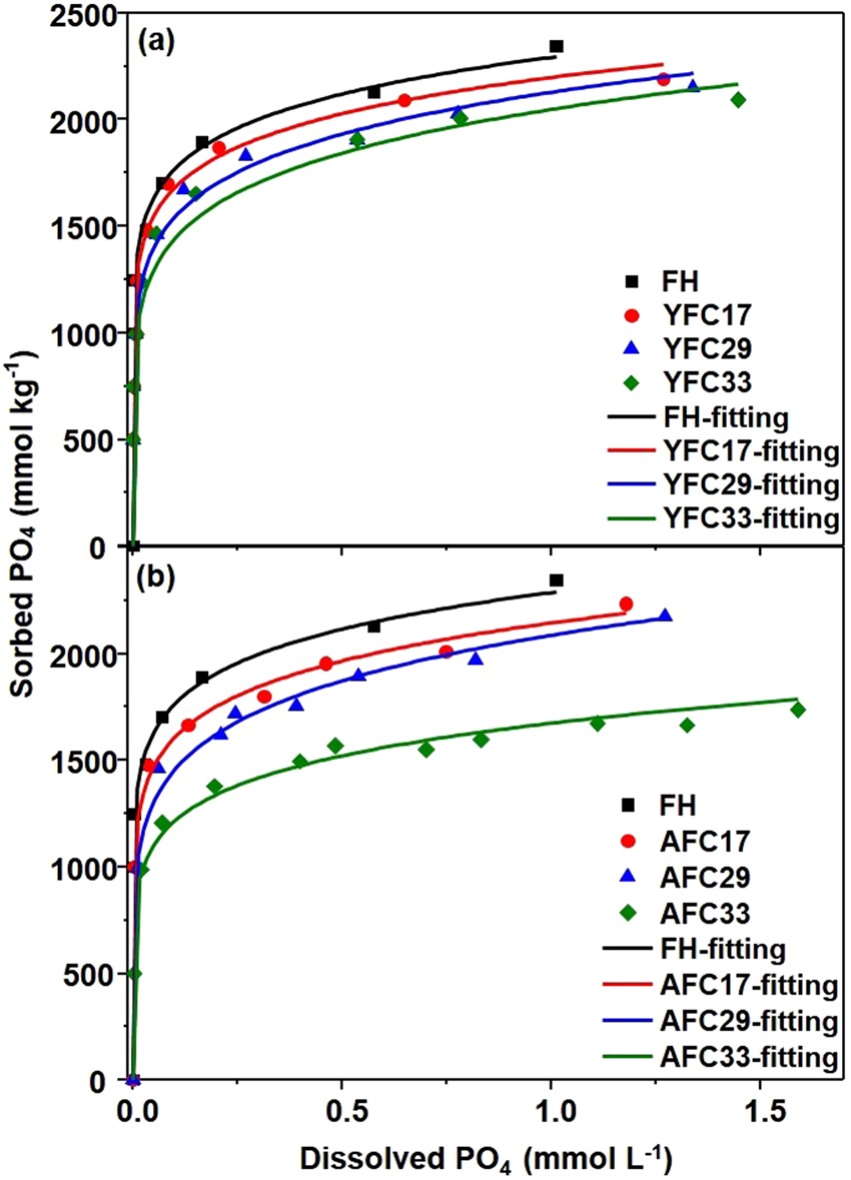
Phosphate adsorption isotherms with Freundlich fitting results (solid lines) for Fe-hydroxide (FH) and HA/Fe coprecipitates containing humic acid: (**a**) YHA and (**b**) AHA with initial C/(C + Fe) ratio of 0.17, 0.29, and 0.33 (YFC17/29/33 and AFC17/29/33).

Figure 5 showed the PO_4_ sorption isotherms on FH and individual YFC and AFC samples along with the Freundlich isotherm models (parameters in Supplementary Table S2). Compared with the maximum observed PO_4_ sorption of 2347 mmol kg^−1^ on FH, the coprecipitation with both HA seemed to decrease the PO_4_ sorption capacity. The maximum observed PO_4_ sorption on YFC samples decreased from 2202 to 2093 as C/(C + Fe) ratios increased from 0.17 to 0.33 (Fig. 5a). On AFC33 sample, however, the maximum observed PO_4_ sorption were only 74% of that on FH (Fig. 5b). It is not surprising to find the decreasing trend in PO_4_ adsorption amounts with increasing C addition. Antelo *et al*.^41^ reported a competition for the adsorption sites on goethite surfaces between HA and PO_4_. Other possible mechanisms accounting for the decreasing PO_4_ adsorption upon the interactions between Fe and negatively-charged HA are charge repulsion, direct site blocking, or aggregation with hydroxide particles^42–44^.

Phosphorus speciation for coprecipitates collected after adsorption isotherms showed that although PO_4_ adsorbed on pure FH dominated the P inventory for all samples, its proportion tended to decrease with increasing C/(C + Fe) ratios (Table 3 and see Supplementary Fig. S4). While all PO_4_ was adsorbed on FH in YFC samples with precipitated C/(C + Fe) ratio $\le$0.29, there was 5.8% of PO_4_ possibly retained by the organically complexed Fe (Fe-peat) via the ternary complex formation on YFC sample with C/(C + Fe) ratio of 0.38^45,46^. In addition, up to 10.8% of P on YFC33 was contributed by the organic form of P, i.e. glycerophosphate that was used as the representative of orthophosphate monoester here and has been often found in HA and humic substances^47^. By contrast, up to 12% of P on AFC samples with precipitated C/(C + Fe) ratio $\ge$0.22 was plausibly contributed by the ternary PO_4_-Fe-OM complexes in which Fe served as bridges between PO_4_ and organic ligands. Collectively, distinct trends in PO_4_ sorption behaviors as the function of C/(C + Fe) ratio between YFC and AFC samples might propose various attributes for surfaces and/or structures of YFC and AFC nanoparticles.

**Table 3:** LCF results for P XANES spectra of HA/Fe coprecipitates containing humic acid (YHA and AHA) with initial C/(C + Fe) ratio of 0.17, 0.29, and 0.33 (YFC17/29/33 and AFC17/29/33) collected after the PO_4_ adsorption^a^^.^

Sample	Adsorbed PO_4_ (mmol kg^−1^)	PO_4_ on ferrihydrite (mol %)	PO_4_ on Fe-peat (mol %)	Glycerophosphate (mol %)	*R-factor*^b^ ( ${\boldsymbol{\times }}$10^3^)
YFC17	2202	100 (0)	0 (0)	0 (0)	4.3
YFC29	2154	100 (0)	0 (0)	0 (0)	4.1
YFC33	2093	83.4 (1.1)	5.8 (1.1)	10.8 (1.5)	0.7
AFC17	2237	100 (0)	0 (0)	0 (0)	5.5
AFC29	2176	84.4 (1.6)	10.7 (1.0)	4.9 (1.4)	0.6
AFC33	1736	68.0 (0.6)	11.6 (0.7)	20.4 (0.7)	2.1

^a^The data show the proportion (in units of mol %) of the reference spectra that resulted in the best fit to the sample data. Numbers in parentheses represent uncertainties in fitting parameters as calculated by Athena. Weighting factors on each fit summed to 100 ± 1 mol %. References used for LCF were PO_4_ adsorbed on ferrihydrite at 600 mmol kg^−1^, PO_4_ adsorbed on Fe(III)-peat coprecipitates at 0.15 mol P mol^−1^ Fe, and commercial β-glycerophosphate diluted in BN at 400 mmol kg^−1^.

^b^Normalized sum of the squared residuals of the fit (*R*-factor = Σ(data-fit)^2^/Σdata^2^).

## Discussion

Coprecipitation between HA and Fe produced PO_4_ adsorption properties that were characteristics of hybridized structures. It has been widely reported that the pre-adsorbed HA on adsorbents such as Fe (hydr)oxides and zerolite reduced the PO_4_ adsorption capacity^43,44,48^. If HA coating on absorbent surfaces was mainly responsible for decreases in PO_4_ adsorption by blocking adsorption sites, changing surface charges, and/or reducing specific surface areas and pore volumes of adsorbents^44,48^, a greater decrease of PO_4_ adsorption should be found for YFC rather than AFC as YFC bore more precipitated C than AFC at the same addition of C/(C + Fe) ratio (Table 1). However, an opposite trend was found in our study. In contrast to significant decreases of PO_4_ adsorption with the increasing C/(C + Fe) ratio on AFC samples (Fig. 5b), differences in PO_4_ adsorption capacity among individual YFC samples seemed relatively indiscernible (Fig. 5a). These results implied that substantial changes in structural development of HA/Fe coprecipitates should be considered for the less PO_4_ bonding sites on AFC samples.

Dissimilar aggregate morphologies between AFC and YFC were evidenced by TEM images (Fig. 2). Results of XRD patterns (see Supplementary Fig. S3) and Fe K-edge EXAFS (Table 2) indicated various degrees of disruption in the short-range structural order of Fe-hydroxide domains between YFC and AFC samples. While stacking disorder of FeO_6_ octahedra for AFC slightly increased with increasing C/(C + Fe) ratio, the greatest structural disordering of FeO_6_ octahedra in YFC occurred at the C/(C + Fe) ratio of 0.20. Most noteworthily, a better developed Fe-hydroxide domain was found in YFC with $\ge$29 C/(C + Fe) mol %. Such discrepancy in structural development between YFC and AFC samples might be plausibly explained by the micellar properties of HA systems^49,50^. While humic negative charges were neutralized by Fe cations during coprecipitation, the expected reorganization of humic networks may cause an overall shrinkage of the humic colloid, leading to the formation of hydrophobic microenvironments referred to intramolecular humic pseudomicelles^50,51^. In YFC17, however, the formation of pseudomicelles might be hindered due to the greatest amount of hydrolyzed Fe relative to the number of humic colloids. Plenty of Fe cations might bridge stretched humic chains, favoring formations of inter-humic clustering^11,52^. Relatively exposed functional groups of YHA could readily react with hydrolyzed Fe(III) and thus interrupt FeO_6_ octahedral linkages. That possibly explained the substantial disintegration of Fe local structures in YFC17 but not in other YFC samples. With the same initial C/(C + Fe) ratio, however, disruptions for FeO_6_ octahedral linkages was hardly found in AFC17. Compared with the YHA composition, AHA contained less amounts of *O*-alkyl, carboxyl, and amine/amide groups (see Supplementary Table S1). Previous studies evidenced that interaction of organic molecules and Fe would be promoted by nitrogen and carbonyl functional groups^17,29^. Associations between carboxyl groups with Fe were found in dissolved OM extracted from fresh litter^15^, peat soils^53^, and suspended matter^52^. Thus, discrepancies in HA/Fe association between YFC and AFC samples suggested the pronounced impact of OM variety on dynamic structural developments of HA/Fe coprecipitates.

Based on Fe EXAFS data and XPS results that showed enrichments of near-surface C relative to Fe, we proposed that AFC consisted of core Fe-hydroxide with surface-outgrowth HA, which is paralleled by its FTIR data that showed AHA-like spectral features (Fig. 1b). While surface-attached AHA bonded to PO_4_ alternatively via ternary PO_4_-Fe-OM complexes (Table 3), it also blocked reactive sites on Fe-hydroxides, resulting in significantly decreasing PO_4_ adsorption with increasing AHA proportion (Fig. 5b). Regarding Fe hydrolysis with HA containing greater proportion of reactive functional groups (YHA), the limited C relative to the hydrolyzed Fe amounts (YFC17) impeded formations of intramolecular humic pseudomicelles, and the HA/Fe association disrupted FeO_6_ linkages. As YHA proportion increased, the partial neutralization of humic macromolecules induced the reorganization of humic networks, leading to a more compact humic network. Such structural shrinkage of YHA possibly lowered the degree of C coverage on FH surfaces, resulting in the decreasing enrichment of near-surface C relative to bulk C with increasing C addition in YFC (Fig. 3). Therefore, domains of FH and YHA might distribute relatively homogeneously in YFC. The FH domains exposed on particle surfaces of YFC yielded similar FTIR spectral features to that of pure FH (Fig. 1) and dominated the PO_4_ adsorption (Table 3), accounting for the macroscale PO_4_ sorption properties of YFC samples nearly equivalent to that of FH (Fig. 5a).

Previous studies reported that C solubilisation from OM/Fe coprecipitates increased with increasing C/(C + Fe) ratios, and up to 3% of Fe released as the Fe(III)-organic complexes when C/(C + Fe) ratios >0.8^15,53^. In our study, however, C solubilisation decreased with increasing C/(C + Fe) ratios and no Fe solubilisation was detected in all samples (Table 1). Different from the water extractable OM in previous studies^15,53^, we used HA that has greater molecular weight and more complicated structure to interact with Fe^54^. We hypothesized that the C stability of HA was controlled by functional groups as they varied structural developments of HA/Fe coprecipitates. In spite of the low C/(C + Fe) ratio, the disordered structure of FH in YFC17 caused by highly associations with polar functional groups of YHA may promote the C solubilisation. In AFC17 that contained the least proportion of carboxyl and *O*-alkyl C, AHA networks that covered the outermost aggregate surfaces may be vulnerable to solubilisation, leading to the greatest C release (19.9%). With increasing C/(C + Fe) ratios, the increasing association between AHA and Fe-hydroxides evidenced by FTIR and XRD results (Fig. 1b and see Supplementary Fig. S3) may alleviate the C solubilisation.

Owing to the importance of FH to segregate PO_4_ and DOM, many studies have determined impacts for the coating of pre-sorbed OM on the PO_4_ adsorption by Fe (hydr)oxides^43,55^. In this study, we evidenced that the diverse range of organic components in HA is one of the keys to affect the structural development of HA/Fe coprecipitates and subsequently the PO_4_ adsorption behavior and C stability. While coprecipitated with HA enriched in polar functional groups, Fe-hydroxides were partially composed of disrupted FeO_6_ octahedral linkages due to the highly association with the functional groups. Structural disruptions of FH showed the promise to enhance the solubilisation for the attached HA. In addition, such Fe domains tended to distribute homogeneously over the coprecipitates with increasing C/(C + Fe) ratios, and the exposed Fe domains on surfaces of HA/Fe coprecipitates were mainly responsible for PO_4_ sorption properties nearly equivalent to that of FH. By contrast, while Fe coprecipitated with HA that possessed limited polar functional groups, the HA/Fe coprecipitates consisted of core Fe-hydroxide domains with surface outgrowth of HA domains. Although the HA networks that were vulnerable to solubilisation enveloped HA/Fe coprecipitates and provided alternative bonding sites for PO_4_ via the ternary complexation with Fe bridges, they would also block reactive sites on FH surfaces, leading to a significant decrease in PO_4_ adsorption (see Supplementary Fig. S5). Recognition of how natural and structurally heterogeneous HA influences the association with Fe hydrolysis could lead to a better estimation for the dynamics of PO_4_, C, and Fe in coprecipitates, resulting in a more precise assessment for the PO_4_ sequestration in wastewater treatment processes and natural aquatic environments.

## Methods

### Synthesis and re-solubilisation of HA/Fe coprecipitates

Tested HA was extracted from an Andisol from the Yangming Mountain in Taiwan (Yangming humic acid, YHA) or purchased from the Sigma-Aldrich (Aldrich humic acid, AHA). See HA preparation in supplementary information. HA was hydrolyzed in 0.01 M KCl solution. Suspensions were adjusted to pH 11.0 and then pH 5.5 using 0.01 M KOH or HCl, shaken for one hour, and passed through 0.2 μm filter membranes. Carbon concentrations in filtrates were determined using the total organic carbon (TOC) analyzer.

The HA/Fe coprecipitates were synthesized via dissolving Fe(NO_3_)_3_•9H_2_O and then pipetting into YHA or AHA suspensions at a point just below the suspension surface under agitation to achieve the Fe concentration of 0.2 mol L^−1^ and the C/(C + Fe) molar ratios of 0.17, 0.29, and 0.33. The HA/Fe coprecipitates containing YHA and AHA were denoted as YFC17/29/33 and AFC17/29/33 hereafter. The YFC and AFC were induced by adjusting suspensions to pH 7.0 with 1.0 M KOH. After vigorously stirred at 250 rpm for 30 min, suspensions were allowed to settle for another 30 min. Coprecipitates were washed three times each with 1 M KCl and 0.01 M KCl by shaking for 30 min and centrifuging. Solids were re-suspended into 0.01 M KCl solution. Stock suspensions were then adjusted to pH 5.5 and stored at 4 °C for a maximum of two weeks. Precipitation of FH without HA addition was also conducted.

Suspensions with a solid concentration of 1.50 g kg^−1^ in a 0.01 M KCl background were used to determine the re-solubilisation of C and Fe from coprecipitates. The pH value for each suspension was maintained at 5.5 over the course of re-solubilisation. After a 42-h incubation, suspensions were centrifuged and passed through a 0.2 μm filter membrane. Concentrations of C and Fe in freeze-dried filtrates were determined (see details in Supplementary information).

### Characterizations of HA/Fe coprecipitates

Subsamples for individual coprecipitates were freeze-dried or preserved as the moist paste for characterizations. Briefly, total C and Fe concentrations were determined. The C and Fe distributions near coprecipitate surfaces, morphologies, and functional groups were determined using XPS, TEM, and FTIR analysis. The XRD patterns collected at the beamline 01C2 of the National Synchrotron Radiation Research Center (NSRRC), Taiwan were used to examine the mineral properties. The Fe K-edge EXAFS analyses performed at the beamline BL-17C1 of the NSRRC were applied to determine the local structures of Fe-hydroxide domains. See details in Supplementary information.

### Phosphate sorption isotherms

Experiments of PO_4_ sorption isotherms were followed the analogous procedures in Liu and Hesterberg^36^. Briefly, the PO_4_ sorption on YFC and AFC samples was conducted at pH 5.5 and 25 ± 1 °C in a 0.01 M KCl background. Various amounts of 0.01 M KH_2_PO_4_ solution were added to YFC or AFC suspensions with the pre-adjusted pH of 5.5 and a solids concentration of 1.50 g kg^−1^ while vigorously stirring. During equilibration, the pH was maintained at pH 5.5 using 0.01 M HCl/KOH. Dissolved PO_4_ in the supernatant solutions was measured using the molybdate colorimetric (Murphy-Riley) procedure. Results of PO_4_ isotherms were fitted using the Freundlich model. Phosphorus speciation for samples containing maximum amounts of adsorbed PO_4_ was determined using the linear combination fitting (LCF) for P K-edge XANES spectra with the end members of β-glycerophosphate and PO_4_ adsorbed on ferrihydrite as well as Fe(III)-peat coprecipitates^56^. See details in Supplementary information.

## Electronic supplementary material

Supplementary Information
