# Supplementary material for: Phosphate Removal in Relation to Structural Development of Humic Acid-Iron Coprecipitates
Source: Sci Rep. 2018 Jul 9;8:10363. doi: 10.1038/s41598-018-28568-7 (PMC6037709; doi:10.1038/s41598-018-28568-7)
Supplement: Supplementary file 1 — Supplementary Information [file 41598_2018_28568_MOESM1_ESM.pdf]

Supplementary information

Phosphate Removal in Relation to Structural Development of Humic Acid-Iron Coprecipitates

Kai-Yue Chen[1], Liang-Ching Hsu[2], Ya-Ting Chan[1], Yen-Lin Cho[1], Fang-Yu Tsao[1], Yu-Min Tzou[1,3], Yi-Cheng Hsieh[4], Yu-Ting Liu[1,3]*

[1] Department of Soil and Environmental Sciences, National Chung Hsing University, 145 Xingda Rd., Taichung 40227, Taiwan.

[2] Scientific Research Division, National Synchrotron Radiation Research Center, 101 Hsin-Ann Road, Hsinchu 30043, Taiwan

[3] Innovation and Development Center of Sustainable Agriculture, National Chung-Hsing University, 145 Xingda Rd., Taichung 40227, Taiwan

[4] Office of the Texas State Chemist, Texas A&M AgriLife Research, Texas A&M University System, College Station, TX 77843, USA

*Corresponding author:
Department of Soil and Environmental Sciences,
145 Xingda Rd., Taichung 40227, Taiwan
Tel: +886-4-22840373 Ext. 3402
Fax: +886-4-22856050
E-mail address: yliu@nchu.edu.tw

Number of Pages – 14
Number of Table – 2
Number of Figures – 5

Methods for Preparation and Characterization

Preparation and characterization of HA

The tested HA was extracted from an Andisol collected at the Yangming Mountain in Taiwan (25°09′N, 121°32′E) (Yangming humic acid, YHA) or purchased from the Sigma-Aldrich (Aldrich humic acid, AHA). Chemical and physical properties for the Andisol and the extraction method for YHA were summarized in previous studies[1,2]. For the AHA, the purification process modified from Kilduff and Weber[3] was conducted prior to the coprecipitation to provide a common ground for the study of individual humic fractions. The AHA suspension was adjusted to pH 11 using NaOH, shaken for 1 hour, and centrifuged at 2857 g for 15 min. The light-colored precipitates were discarded. Humic materials remaining in suspensions were then precipitated using 1 M HCl. After the acidification, suspensions were centrifuged again, and the absorbance at 254 nm of supernatant was measured to determine the remaining amount of fulvic acid. This purification process was repeated until the 254-nm absorbance kept constant. Final precipitates were washed three times each with 1 M KCl and 0.01 M KCl solutions by shaking for 30 min and centrifuging for 15 min to finish the purification. The extracted YHA and purified AHA was lyophilized and stored in a desiccator until use. The C content, elemental analysis, ^{13}C nuclear magnetic resonance (NMR) spectra, Fourier transform infrared (FTIR) spectra, and transmission electron microscopy (TEM) images were analyzed for characterization. See details below.

Total Concentration of C and Fe

Freeze-dried subsamples were dissolved in concentrated HNO_3. Concentrations of C and Fe were determined using a total organic carbon (TOC) analyzer (analytikjena multi N/C 2100) and inductively coupled plasma atomic emission spectroscopy (ICP-AES, Spectro Genesis), respectively.

Elemental analysis

Subsamples were freeze-dried for 2 h prior to the determination for contents of C, H, N, and O performed by the Heraeus CHN-O-S Rapid Analyzer. For the O analysis, 5 mg of subsample was burned in a pyrolysis tube containing platinized carbon. The non-dispersive infrared sensor detector was used to measure CO or CO_2 for the calculation of O contents. For C, H, and N analyses, the combustion of 3.5 mg subsample was conducted in a vertical tube at 1800℃ under a high purity oxygen environment. Concentrations of C, H, and N were calculated based on concentrations of CO_2, H_2O and N_2 detected by a thermal conductivity detector.

^{13}C Nuclear magnetic resonance (NMR)

An aliquot of 300 mg subsample was spun at a magic angle spin rates of 6.5 kHz using a Bruker DSX400WB NMR spectrometer. For each scan, a pulse with a carrier frequency of 100 MHz was applied and the contact time was 15 μs. In total, 2168 scans were acquired for each spectrum.

Fourier transform infrared (FTIR) spectroscopy

Two mg of freeze-dried subsample was mixed with 200 mg of KBr and compressed into a translucent disk. For each samples, 256 individual scans in the range of 4000 – 400 cm^{-1} were acquired in the transmission mode with an optical resolution of 4 cm^{-1} using a Thermo Nicolet Nexus 470 FTIR spectrometer.

Transmission electron microscopy (TEM)

For TEM analysis, suspensions were prepared by mixing subsamples with a hundredfold volume of deionized water and then evaporated on C-coated films supported by copper grids. Microscale morphologies of dispersed particles were obtained using the JEOL JEM-2010.

X-ray photoelectron spectroscopy (XPS)

The XPS analyses were performed using a ULVAC-PHI, PHI 5000 Versa Probe. Surface concentrations of C, O, and Fe (in atom percent) were obtained from spectral deconvolution and fitting of C 1s, O 1s, and Fe $2p_{3/2}$ signals.

X-ray diffraction (XRD)

The XRD patterns were obtained using a synchrotron-based XRD instrument at the beamline 01C2 of the National Synchrotron Radiation Research Center (NSRRC), Taiwan. Subsamples were mounted in metal sample holders. Diffraction patterns were recorded with a 300 s exposure time in the 2 theta range from 5 to 40° at the energy of

16 keV, wherein the corresponding wavelength of the incident X-ray was 0.77 Å.

Fe K-edge extended X-ray absorption fine structure (EXAFS) spectroscopy

The Fe K-edge EXAFS spectrum was collected at the beamline BL-17C1 of the NSRRC, which is equipped a Si(111) monochromator. Moist pastes of subsamples were mounted in acrylic sample holders and covered with Kapton tape. Spectra were acquired in transmission mode using a $N_{2(g)}$-filled ion chamber detector in the photon energy ranging from -200 to 700 eV relative to the Fe absorption edge of 7112 eV. The step size was 0.5 eV between -30 to +50 eV and 0.05 $Å^{-1}$ for higher energies. Details for data analysis were similar as that reported in our previous study[4]. Briefly, at least four scans of each sample were aligned, merged, and processed using the IFEFFIT interface including the Athena and Artemis programs[5].

For the fitting of Fe K-edge EXAFS data, the structural model of two-line ferrihydrite was used to calculate the single scattering Fe-O and Fe-Fe paths out to a maximum radial distance of 3.5 Å[6]. Given that samples for EXAFS analysis were prepared as moist pastes, individual Fe atoms are expected to be coordinated with six O atoms, including surface water molecules, regardless of surface defects[7]. The edge- and corner-sharing FeO_6 linkages were fitted using the paths of Fe-Fe1 and Fe-Fe2 at 3.02 and 3.40 Å. Structural parameters including coordination number (CN), interatomic distance (ΔR), and mean-square displacement of interatomic distance (σ^2) were fitted for

all EXAFS spectra. The amplitude reduction factor (S_0^2) was derived from first-shell fitting of hematite and fixed at 0.83.

Phosphorus K-edge X-ray absorption near edge structure (XANES) spectroscopy

About 0.2 g of the moist solids was mounted in acrylic sample holders and covered with 5μm polypropylene X-ray film (Spex Industries, Edison, NJ) to inhibit desiccation. Phosphorus K-edge XANES data were collected at Beamline BL16A1 of the NSRRC. The synchrotron radiation was detuned by 10% at 200 eV above the P(V) K-edge. Spectra between -50 to +250 eV relative to the P(V) K-edge energy at 2151 eV were collected in fluorescence mode in a $N_{2(g)}$-filled Lytle detector, using a step size of 0.1 eV across the absorption edge region (-5 to +10 eV). The nearly consistent intensity for the white-line peaks suggested indiscernible self-absorption effects[8].

Collected spectra were merged, baseline corrected, and normalized using similar approach described above for Fe EXAFS analysis. The P speciation was determined using linear combination fitting (LCF) analyses across the absorption edge from -20 to 30 eV using the Athena program[5]. The P XANES spectra for PO_4 adsorbed on ferrihydrite at 600 mmol kg^{-1}, PO_4 adsorbed on Fe(III)-peat coprecipitates at 0.15 mol P mol^{-1} Fe[9], and commercial β-glycerophosphate (Sigma-Aldrich) diluted in BN at 400 mmol kg^{-1} were also collected and used as end-member standards in LCF analyses.

Table S1. The elemental composition, integrative result of NMR spectrum, and polarity for Yangming humic acid (YHA) and Aldrich humic acid (AHA).[a]

Sample	Elemental composition (%)				mole kg^{-1}	Distribution of chemical shift (%)						
						0-50 ppm	50-112 ppm	112-145 ppm	145-163 ppm	163-190 ppm	190-215 ppm	
	C	H	O	N	Fe	alkyl C	*O*-alkyl C	aromatic C	phenolic C	carboxyl C	carbonyl C	Polar C[b]
YHA	44.4 (0.11)	4.2 (0.09)	38.2 (0.02)	2.0 (0.02)	0.07 (0.003)	28.7	29.3	26.6	3.8	10.5	1.2	58.0
AHA	49.6 (0.12)	4.3 (0.13)	39.6 (0.07)	0.7 (0.02)	0.08 (0.005)	36.5	13.3	33.0	7.5	7.7	2.0	49.8

[a] Numbers in parentheses represent standard deviations derived from replicates.
[b] Polar C: total polar C region of 50–112 and 145–215 ppm[2].

Table S2. Freundlich parameters for adsorption isotherms of PO_4 on Fe-hydroxide (FH) and HA/Fe coprecipitates containing Yangming humic acid (YHA) and Aldrich humic acid (AHA) with initial C/(C+Fe) molar ratio of 0.17, 0.29, and 0.33 (YFC17/29/33 and AFC17/29/33).[a]

	Freundlich isotherm		
sample	K_d	1/*n*	R^2
FH	2290.9	0.11	0.995
YFC17	2197.9	0.12	0.984
YFC29	2130.1	0.14	0.980
YFC33	2048.3	0.15	0.998
AFC17	2143.4	0.13	0.988
AFC29	2087.4	0.16	0.971
AFC33	1677.6	0.14	0.943

[a] Freundlich model is expressed as $q = K_d C^{1/n}$, where *q* is the amount of adsorption, *C* is the equilibration adsorptive concentration, K_d is the distribution coefficient, and *n* is a correction factor.

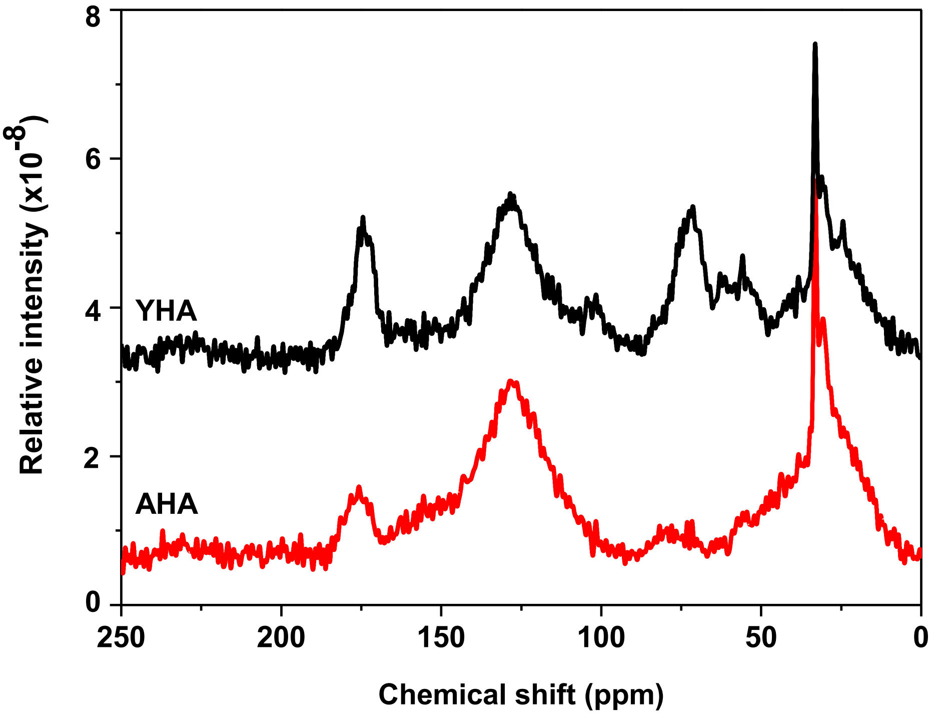

Figure S1. The ^{13}C NMR spectrum of Yangming humic acid (YHA) and Aldrich humic acid (AHA).

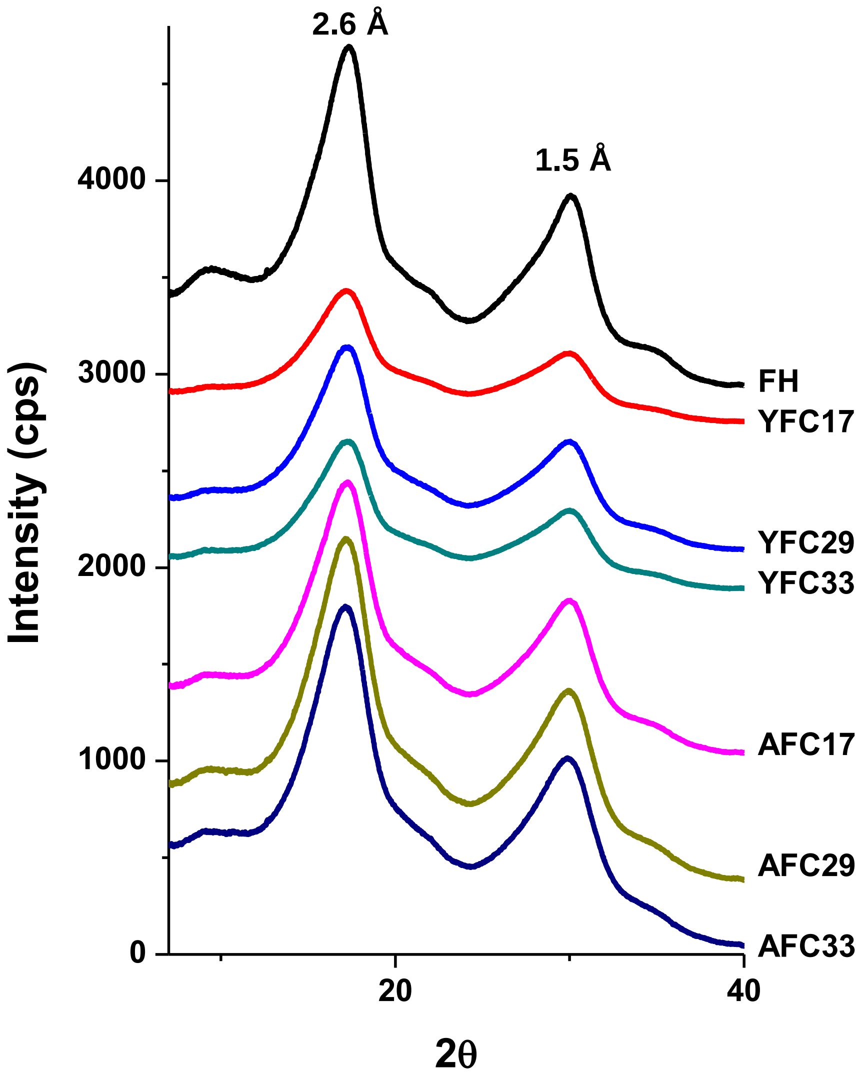

Figure S2. XRD patterns of Fe-hydroxide (FH) and HA/Fe coprecipitates containing humic acid (YHA and AHA) with initial C/(C+Fe) ratio of 0.17, 0.29, and 0.33 (YFC17/29/33 and AFC17/29/33).

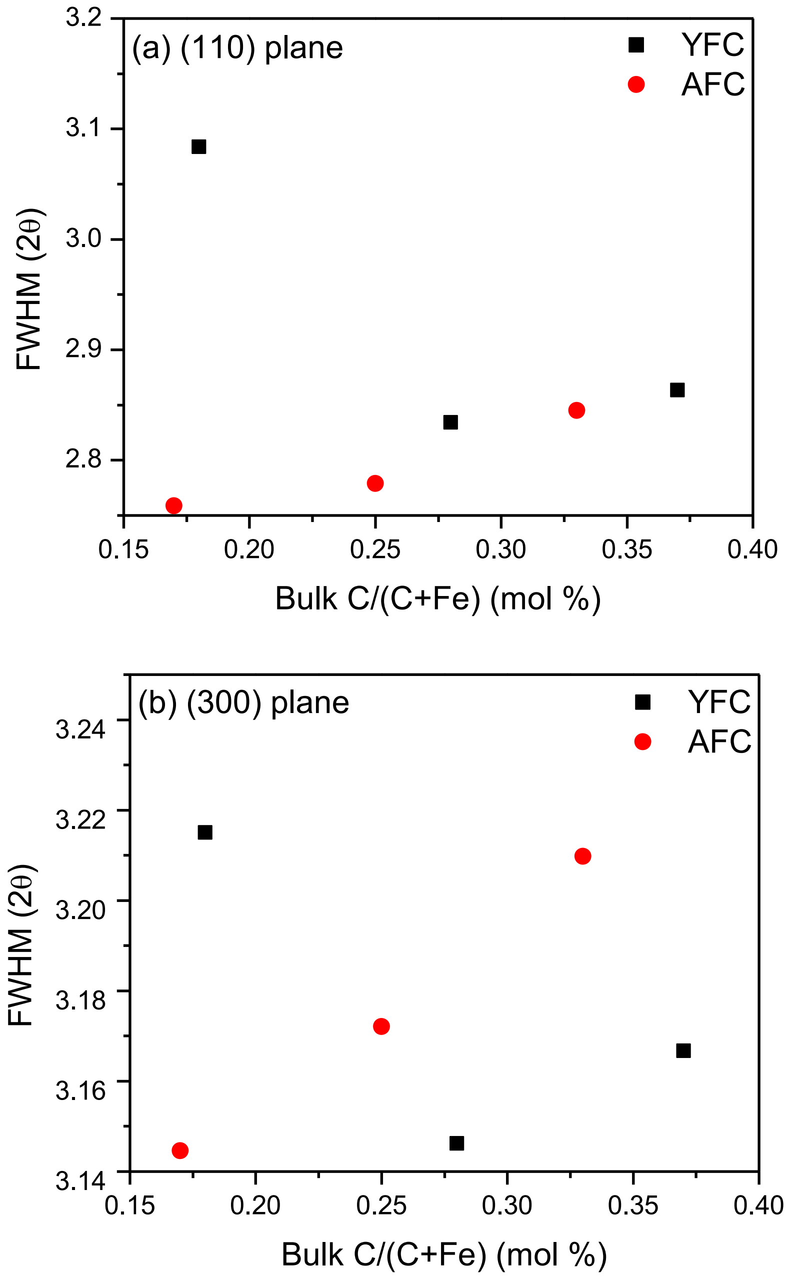

Figure S3. Full width at half maximum of the (a) (110) and (b) (300) planes in XRD data for Fe coprecipitated with Yangming humic acid (YFC) and with Aldrich humic acid (AFC).

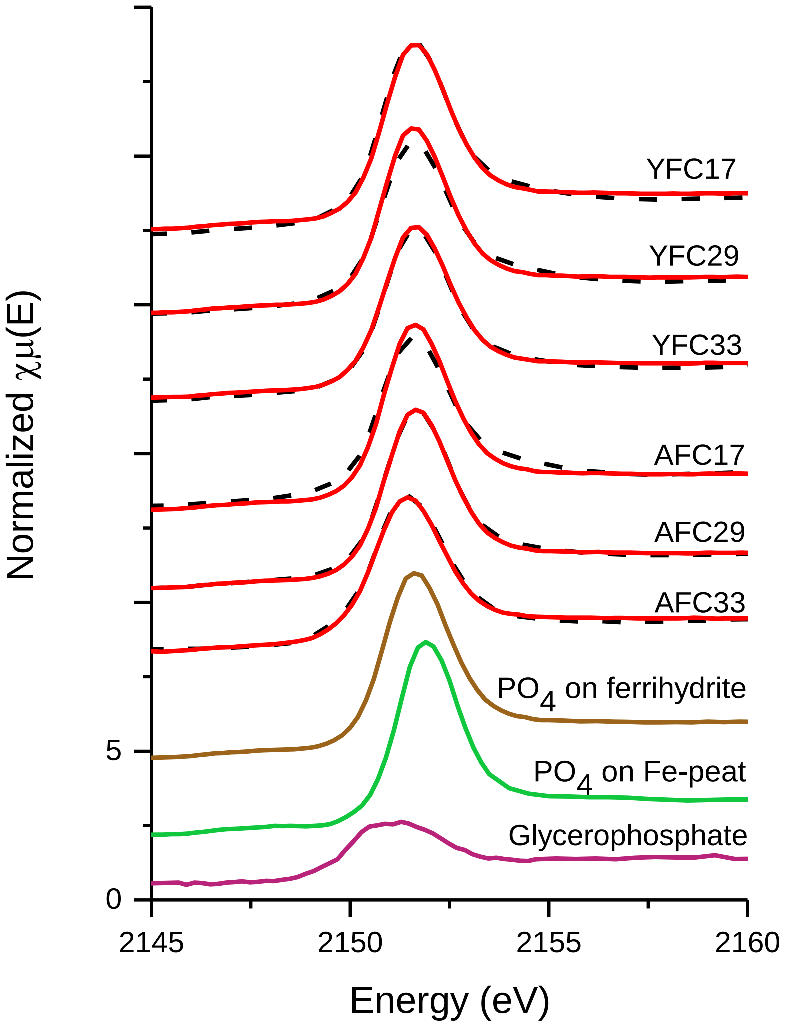

Figure S4. Phosphorus K-edge XANES spectra (dashed lines) and results of linear combination fitting (LCF - solid lines) for HA/Fe coprecipitates as well as spectra for end-member standards of PO_4 adsorbed on ferrihydrite, PO_4 adsorbed on Fe(III)-peat coprecipitates, and β-glycerophosphate. The HA/Fe coprecipitates collected after the PO_4 adsorption contain Yangming humic acid (YHA) and Aldrich humic acid (AHA) with initial C/(C+Fe) molar ratio of 0.17, 0.29, and 0.33 (YFC17/29/33 and AFC17/29/33). The adsorbed PO_4 concentrations were 2202, 2154, 2093, 2237, 2176, and 1736 mmol kg^{-1} on YFC17/29/33 and AFC17/29/33 samples, respectively.

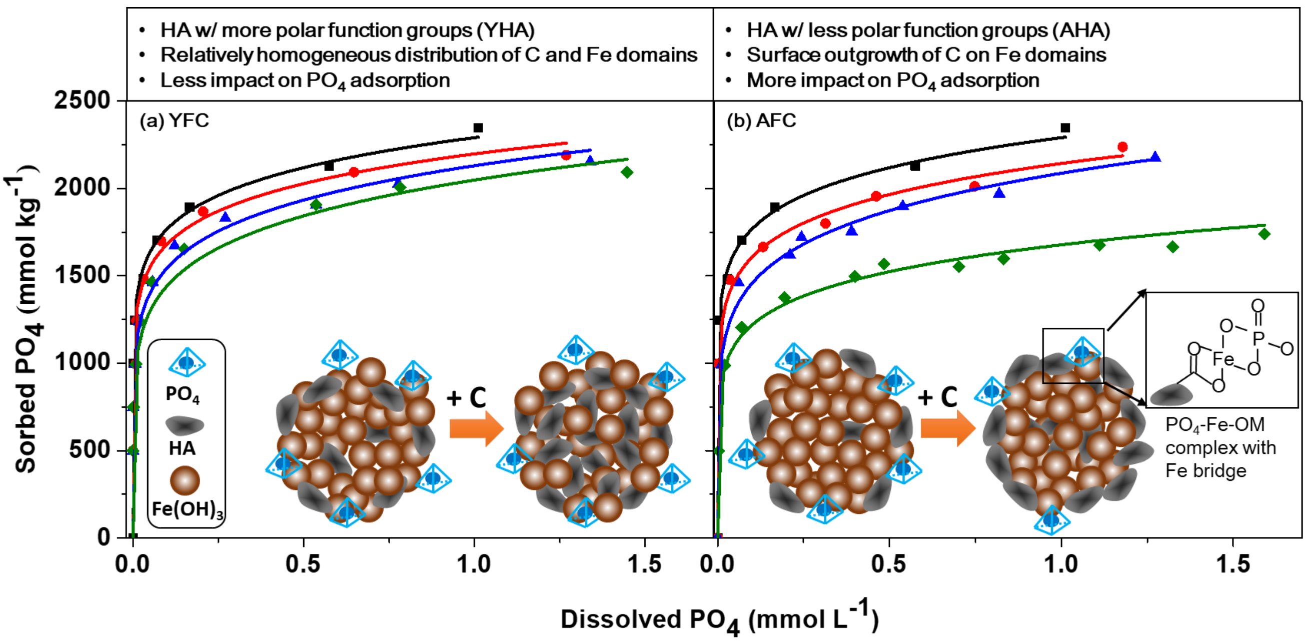

Figure S5. Diagrammatic sketch for sorption mechanisms of phosphate on (a) YFC and (b) AFC (HA/Fe coprecipitates containing YHA and AHA, respectively). Although HA networks that enveloped HA/Fe coprecipitates provided alternative bonding sites for PO_4 via the ternary complexation with Fe bridges (showed as the expanded view in the inset of Fig. S5b), they would also block reactive sites on surfaces of Fe-hydroxides.

References

1 Stevenson, F. J. *Humus Chemistry: Genesis, Composition, Reactions*. (John Wiley & Sons, INC, 1994).

2 Huang, Y. Y. *et al.* Influences of preparative methods of humic acids on the sorption of 2,4,6-trichlorophenol. *Chemosphere* **70**, 1218-1227, doi.org/10.1016/j.chemosphere.2007.08.052 (2008).

3 Kilduff, J. & Weber, W. J. Transport and separation of organic macromolecules in ultrafiltration processes. *Environ. Sci. Technol.* **26**, 569-577, doi: 10.1021/es00027a021 (1992).

4 Liu, Y. T. & Hesterberg, D. Phosphate Bonding on Noncrystalline Al/Fe-Hydroxide Coprecipitates. *Environ. Sci. Technol.* **45**, 6283-6289, doi: 10.1021/es201597j (2011).

5 Ravel, B. & Newville, M. ATHENA, ARTEMIS, HEPHAESTUS: data analysis for X-ray absorption spectroscopy using IFEFFIT. *J. Synchrotron Radiat.* **12**, 537-541, doi.org/10.1107/S0909049505012719 (2005).

6 Michel, F. M. *et al.* The structure of ferrihydrite, a nanocrystalline material. *Science* **316**, 1726-1729, doi: 10.1126/science.1142525 (2007).

7 Manceau, A. & Gates, W. P. Surface structural model for ferrihydrite. *Clays Clay Miner.* **45**, 448-460 (1997).

8 Khare, N., Hesterberg, D., Beauchemin, S. & Wang, S. L. XANES determination of adsorbed phosphate distribution between ferrihydrite and boehmite in mixtures. *Soil Sci. Soc. Am. J.* **68**, 460-469, doi:10.2136/sssaj2004.4600 (2004).

9 Morris, A. J. & Hesterberg, D. L. Iron (III) coordination and phosphate sorption in peat reacted with ferric or ferrous iron. *Soil Sci. Soc. Am. J.* **76**, 101-109, doi:10.2136/sssaj2011.0097 (2012).
